# Predicting airborne coronavirus inactivation by far-UVC in populated rooms using a high-fidelity coupled radiation-CFD model

**DOI:** 10.1038/s41598-020-76597-y

**Published:** 2020-11-12

**Authors:** Andrew G. Buchan, Liang Yang, Kirk D. Atkinson

**Affiliations:** 1 School of Engineering and Materials Science, Queen Mary University of London, London, E1 4NS UK; 2 School of Water, Energy and Environment (SWEE), Cranfield University, Bedford, MK43 0AL UK; 3 Faculty of Energy Systems and Nuclear Science, Ontario Tech University, Oshawa, Ontario L1G 0C5 Canada

**Keywords:** SARS-CoV-2, Viral infection, Mathematics and computing

## Abstract

There are increased risks of contracting COVID-19 in hospitals and long-term care facilities, particularly for vulnerable groups. In these environments aerosolised coronavirus released through breathing increases the chance of spreading the disease. To reduce aerosol transmissions, the use of low dose far-UVC lighting to disinfect in-room air has been proposed. Unlike typical UVC, which has been used to kill microorganisms for decades but is carcinogenic and cataractogenic, recent evidence has shown that far-UVC is safe to use around humans. A high-fidelity, fully-coupled radiation transport and fluid dynamics model has been developed to quantify disinfection rates within a typical ventilated room. The model shows that disinfection rates are increased by a further 50-85% when using far-UVC within currently recommended exposure levels compared to the room’s  ventilation alone. With these magnitudes of reduction, far-UVC lighting could be employed to mitigate SARS-CoV-2 transmission before the onset of future waves, or the start of winter when risks of infection are higher. This is particularly significant in poorly-ventilated spaces where other means of reduction are not practical, in addition social distancing can be reduced without increasing the risk.

## Introduction

The coronavirus pandemic has put hospitals and long term care facilities under considerable stretch. Aerosolised coronavirus released through breathing was probably a significant cause of this^1,2^. In these environments, and some other populated spaces, social distancing may be impractical and hence the infection controls must focus on a combination of personal hygiene and correct use of personal protective equipment (PPE). With major shortages seen in many countries, most visibly the supply of N95 face masks^3^, availability of adequate PPE has remained a major concern throughout the crisis. As many countries exit their lockdowns, fatigue and habituation within the population may lead to increased complacency in hygiene measures, and hence, along with reducing the burden on PPE, controls like ultraviolet germicidal irradiation^4^ (UVGI) have been considered. UVGI has previously been considered as a way of controlling airborne viruses during a pandemic if effective vaccines or antiviral drugs are not available^5^. Used for over a hundred years, UVGI-based disinfection traditionally relies on cancer-causing 254 nm UVC light thereby rendering it incompatible for use around people. Fortuitously, recent advances in UV lamp technology, in particular excimer lamps^6–8^ and light-emitting diodes^9–11^, now permit narrow bandwidth, short wavelength UVC (207–222 nm) to be generated. As these far-UVC wavelengths cannot penetrate either the human stratum corneum or ocular tear layer^12^, they are not carcinogenic or cataractogenic^13–17^ and can therefore be safely used in people-facing applications^18^.

Quantifying the rate of far-UVC viral inactivation within a general room is complex and multiphysics in nature. It requires both radiation and atmospheric flow calculations where objects within rooms add complication as they obstruct both the light propagation and air flows, thus casting shadows and inducing eddies and turbulent structures. High fidelity modelling is therefore essential, and here we present the first coupled radiation transport and fluid dynamics simulator, based on the Boltzmann Transport and Navier–Stokes equations with integrated Large Eddy Simulation (LES) turbulence models, for viral inactivation within atmospheres. Fully resolved spatially distributed far-UVC intensities enable more accurate predictions of virus removal over simplified $1/r^2$ strategies^19^, diffusion radiation models^20^, and, potentially, empirical data taken from physical measurements^21–23^. The use of LES models^24^ provide more detailed descriptions of viral transport over other modelling methods, such as Reynolds Averaged Navier–Stokes^21,23^ or analytical zone-mixing methods^23,25^, and despite their increased computational requirement, and hence limited use, their importance is now being recognised in the field of atmospheric viral transport predictions^24^.

**Figure 1:**
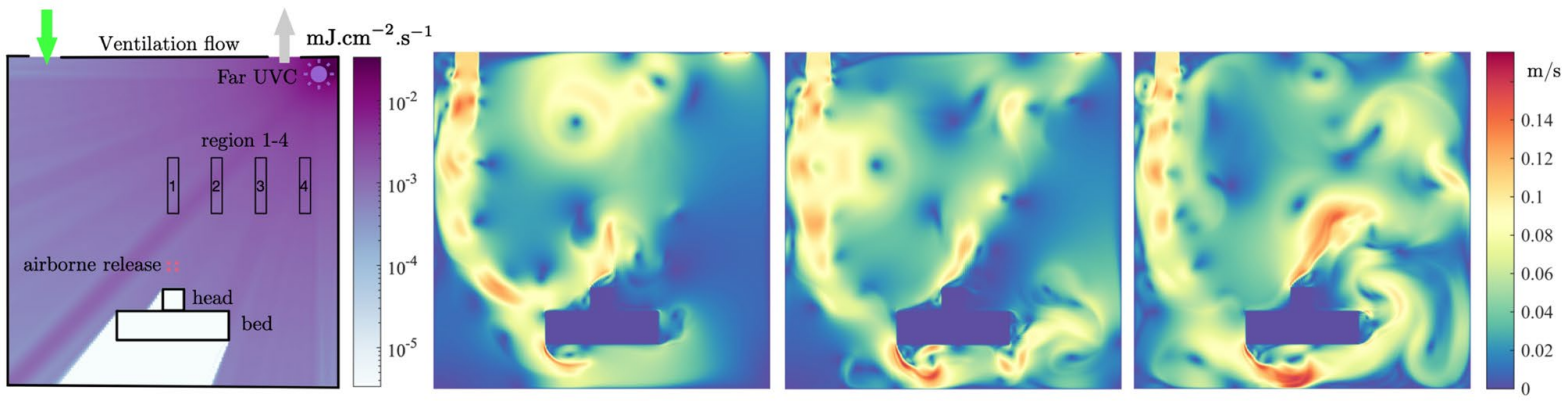
Left to right: Two-dimensional hospital or care home room with bed and patient regions with superimposed far-UVC intensity field (units ${\text {mJ}}.{\text {cm}}^{-2}. {\text {s}}^{-1}$): Flow velocity profiles at 10, 50 and 100 s following viral release.

This model was used to study the far-UVC inactivation of aerosolised human coronavirus in a single occupancy private room, a representative environment found in hospitals and long-term care facilities. Conducted in the two-dimensional domain shown in figure 1, the room was of $3\,{\text {m}}$ by $3\,{\text {m}}$ cross-section and occupied by a patient laying in a bed. The room was air conditioned with inlet and outlet vents located in the top left and top right regions of the ceiling, respectively. Two inlet air velocities, $0.1\,{\text {m}}{\text {s}}^{-1}$ and $0.01\,{\text {m}}{\text {s}}^{-1}$ were analysed. The resulting air changes per hour (ACH) were 8.0 and 0.8, respectively. A $0.1\,{\text {m}}$ by $0.1\,{\text {m}}$ region above the patient serves as the source zone for virus exhaled by the patient. The viral load expelled into the room was modelled in two forms. First was a single 2 s pulse with normalised density of $1 \,\hbox {pfu}.{\text {s}}^{-1}$ representing a single unobstructed breath. The second was a series of 2 s pulses with normalised density of $1 \,\hbox {pfu}.{\text {s}}^{-1}$, separated by 2 s pauses, representing continuous unobstructed breathing. In all calculations, flow fields were allowed to develop by simulating the air conditioning system for 100 s before viral release was activated in the source zone. Transport and concentration of coronavirus was simulated for a further 2400 s, taking into consideration evolving flow fields, removal from the outlet vent, inactivation due to far-UVC exposure, and natural losses due to the biological half life of approximately 1.2 h in aerosols^26^. The source of far-UVC originated from a lamp positioned in the top right corner of the room. The power investigated yielded far-UVC intensities of approximately $0.0009\,{\text {mJ}}.{\text {cm}}^{-2}. {\text {s}}^{-1}$ over the region occupied by the patient, and 0.0007-$0.0014\,{\text {mJ}}.{\text {cm}}^{-2}. {\text {s}}^{-1}$ at head-height (standing) regions depending on the proximity to the far-UVC lamp. These are close to the currently recommended exposure limit^12,27^. A far-UVC inactivation value of $Z=4.1\,{\text {cm}}^2.{\text {mJ}}^{-1}$ for human coronavirus was used, based on the most recent estimates and is considered representative of SARS-CoV-2^12^.

### Results

The spatially varying intensity of the far-UVC field produced by the lamp is presented in Fig. 1. The employment of a full Boltzmann solver to resolve the radiation intensity provides an accurate description across all space. Here the solution exhibits the typical drop off of intensity away from the lamp, and accounts for removal due to interactions with air and the shadows formed from the presence of solid objects.

This radiation field is considered constant in time and is used in all subsequent analysis. Figure 1 also presents the flow velocities at 3 time instances of 10, 50 and 100 s following the viral release. The flow fields have evolved into a quasi-steady state, rotating anti-clockwise, with eddies forming due to the presence of the patient and the bed.

**Figure 2:**
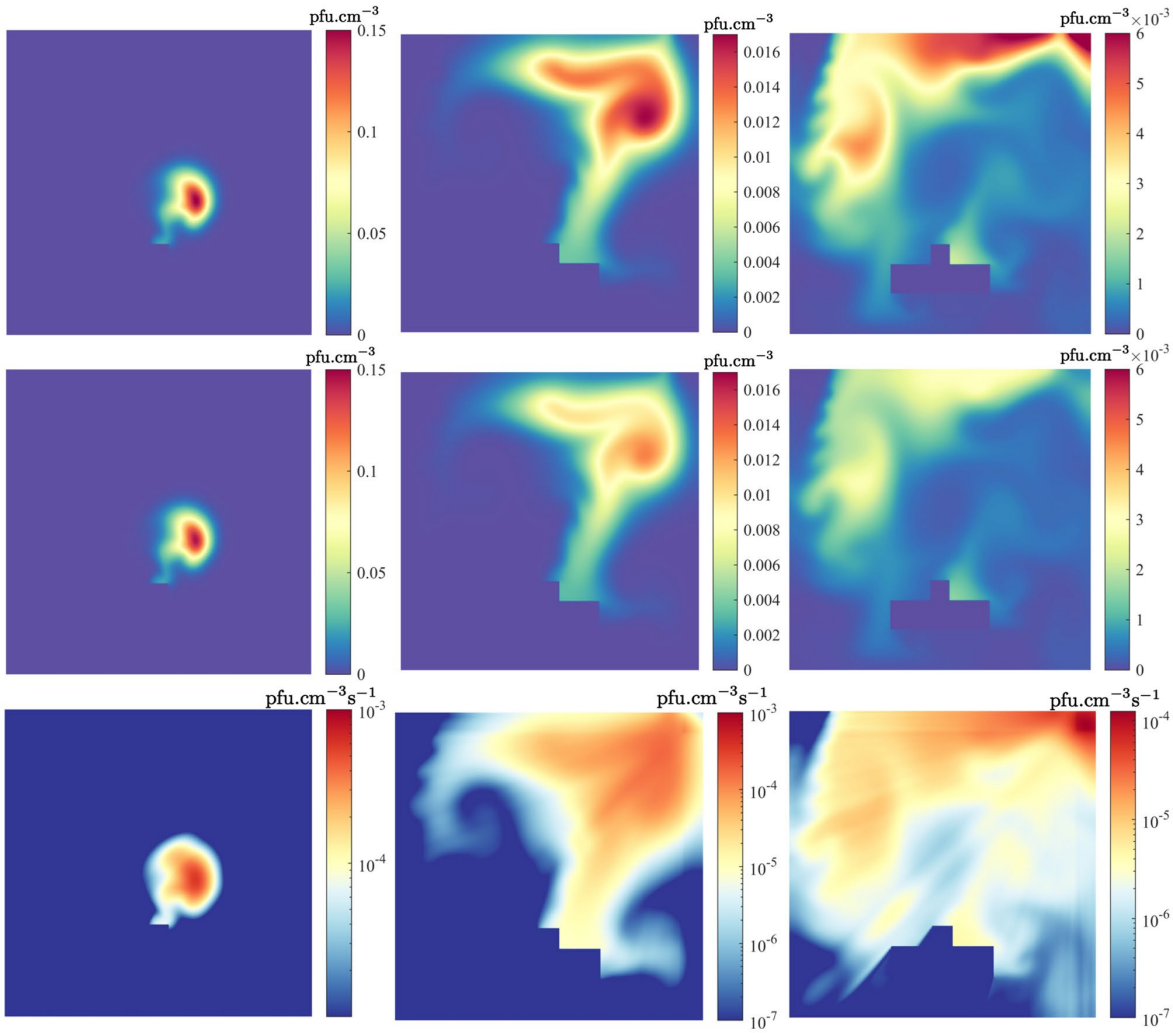
Left to right: Solution profiles at 10, 50 and 100 s after release, with 8.0 ACH ventilation. Top row: Viral distribution without far-UVC. Middle row: Viral distribution with far-UVC, Bottom row: rate of viral inactivation.

**Figure 3:**
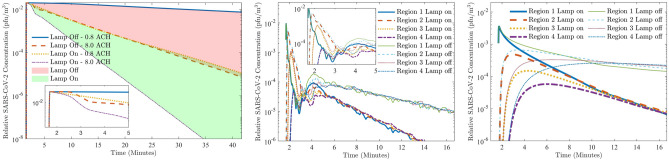
Left to right: Virus concentration in (**a**) whole room; (**b**) regions with 8 ACH; (**c**) regions with 0.8 ACH.

Figure 2 shows the viral distributions resulting from the single pulse of SARS-CoV-2 at 10, 50 and 100 s (from viral release) with and without far-UVC light. Apart from reducing peak concentrations, a notable feature is the sharp viral reduction in the vicinity of the lamp which, under this setup, has prevented some of its re-circulation. This is highlighted by the removal rates presented in the figure; large reductions being seen in the upper regions of the room, whilst small reductions are found where far-UVC shading is present. The graphs presented in Fig. 3a compare the room’s total viral concentration over time. Without the lamp, 0.8 ACH ventilation results in very slow reductions, but when increased to 8.0 ACH, viral removal through ventilation begins 45 s after release and concentrations are reduced by 90% and 99% in approximately 12 and 24 minutes, respectively. By coincidence, near identical reduction times were observed when using far-UVC in combination with 0.8 ACH ventilation, here again taking 12 and 24 minutes, respectively. The combination of far-UVC and high ventilation reduces the viral count most effectively, times to achieve 90% and 99% reductions being approximately 6 and 11.5 minutes, respectively, more than halving the times when using 8.0 ACH ventilation alone. Figure 3b,c present the viral concentrations in the 4 regions outlined in Fig. 1. The highest viral concentrations occur across the regions closest to the bed soon after release where the concentrations spike due to their downwind positions from the source. Secondary spikes are also observed as the viral plume, which has yet to fully dissipate, circulates the room and re-enters the monitored regions. However, viral levels over all regions converge to similar quantities after about 5 and 12 minutes with 8.0 and 0.8 ACH ventilation, respectively, indicating the time taken for the localised viral release to mix homogeneously throughout the room. The use of far-UVC results in faster removal of virus at all distances. As before, with 8.0 ACH, the lamp reduces the time for similar reductions by more than half. For 0.8 ACH ventilation, given that the viral concentration plateaus without the lamp, reduction times are significantly greater.

**Figure 4:**
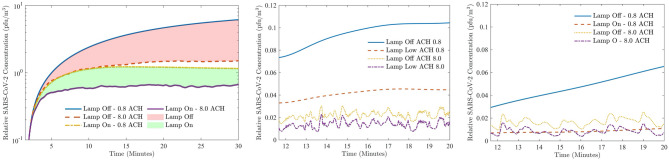
Left to right: Virus concentration in (**a**) whole room; (**b**) region 2; (**c**) region 4.

The graphs presented in figure 4 show viral concentrations resulting from the source from a repeated series of 2 s exhalations. Figure 4a presents the total viral concentration within the room over time. With 0.8 ACH ventilation and no far-UVC sterilization, the viral concentration rises steadily for the duration of the simulation. When increasing the ventilation to 8.0 ACH, the viral concentration stabilises within 18 minutes without far-UVC. By comparison, with 8.0 ACH ventilation, the viral concentration with far-UVC also stabilises, but their numbers are reduced by a further 57%. Furthermore, when used in combination with 0.8 ACH ventilation, the far-UVC is still more effective that 8.0 ACH ventilation alone, where the additional reduction in viral concentration is approximately 20%. Importantly, comparing the use of far-UVC with low 0.8 ACH ventilation shows the reduction in viral concentration is approaching an order of magnitude, i.e. a 90% level. At the end of the simulation the reduction was of the order of 85%, however the viral concentration was continuing to rise without the far-UVC, thus the indication is that reductions will continue to grow over longer timescales.

Figure 4b,c present the viral concentrations in regions 2 and 4. The SARS-CoV-2 levels are highest closer to the viral source, but reductions are observed using far-UVC. With 8 ACH ventilation the far-UVC reduces the concentrations in regions 2 and 4 by a further 40% and 52%, respectively. For the lower 0.8 ACH ventilation, the additional reductions over ventilation increase to 58% and 85%, respectively. Interestingly, with 8 ACH ventilation, the average SARS-CoV-2 concentration in region 2 with far-UVC is around 24% lower than in region 4 without far-UVC. With 0.8 ACH this increases to 42%. This is despite the distance to the viral source being reduced from 1.25 m to 0.5 m.

## Discussion

A plethora of approaches are being used to mitigate transmission of aerosolised SARS-CoV-2 coronavirus. Others are proposed. Most of these follow one or more of three key principles: minimise time exposed to the virus (limit interactions), maximise distance from sources of virus (social distancing), or shield yourself from the virus (wear PPE). Whilst these are all effective measures, their success is tied to human behaviour and hence at risk from complacency. Unlike these active measures, passive use of in-room far-UVC provides an invisible barrier. Whilst the viability of human coronaviruses can be successfully reduced by far-UVC^12^, we have shown that the contention that it can be reduced by 99.9% in public spaces within 25 minutes^12^ is situation dependent. In a representative environment in a hospital or a long-term care facility, the nature of the viral source and the interaction of ventilation with far-UVC illumination all strongly influence the efficacy of far-UVC germicidal irradiation.

For poor ventilation and far-UVC human exposures at the currently recommended level, total viable viral concentration is reduced exponentially in comparable times to those previously stated^12^. However, it has been shown that this is only the case for a single seeding of virus particles such as those which occur from a single unobstructed breath. Such rapid reductions could therefore be achieved in situations where face masks or breathing apparatus are removed for a short period of time. Given the normal pattern of unobstructed human breathing constantly seeds a poorly-ventilated room with new virus, concentrations ultimately reach an equilibrium. With far-UVC illumination at currently recommended exposure levels, not only is this equilibrium reached more quickly, but the viral concentration is approximately one order of magnitude lower than it would be without. In highly-ventilated rooms, reductions in in-room viral concentration for both breathing scenarios are comparable to those from far-UVC at currently recommended exposure levels in poorly-ventilated rooms. Even in highly-ventilated rooms where satisfactory levels of removal may already exist, far-UVC illumination will further reduce viral concentrations by around 57%.

Several practical implications of far-UVC illumination in reducing in-room transmission of SARS-CoV-2 are clear. Firstly, with both high and low ventilation, far-UVC will reduce aerosolised SARS-CoV-2 concentrations within a metre of the patient to levels below that in regions beyond a metre without far-UVC. Employment of far-UVC could therefore have a bearing on the social distancing limits currently used in many countries, or at least further reduce risks of transmission at these distances. Secondly, in all scenarios described, far-UVC will reduce in-room SARS-CoV-2 concentrations to levels comparable to that provided practically by breathing through an N95 mask^28,29^. Finally, unlike face masks, far-UVC is a passive control from the perspective of the individual. Due to it having a similar efficiency to an N95 mask, it could replace them in some situations, reducing the demand for PPE supplies, and lessening the damage that PPE disposal is causing to the environment^30^.

## Methods

The survival rate *S* of a viral population subjected to some UVC radiation intensity over a time period of *t* seconds is governed by the equation,

1 $$\begin{aligned} S =e^{-Zd} =e^{-ZE_pt}, \end{aligned}$$

as described in^4^. The UVC intensity with dimension ${\text {mJ}}.{\text {cm}}^{-2}. {\text {s}}^{-1}$ is denoted by $E_p$, and the dose received (with units ${\text {mJ}}.{\text {cm}}^{-2}$) is denoted by $d = E_p t$. The key parameter governing the rate of viral inactivation is the susceptibility value Z, with units ${\text {cm}}^2.{\text {mJ}}^{-1}$. This susceptibility value is dependent on both the virus type and its hosting media. Relating to SARS-CoV-2 estimates of *Z* have been provided in^12^ which states a value $4.1\,{\text {cm}}^2.{\text {mJ}}^{-1}$ for moist air conditions.

### Far-UVC radiation transport model

The intensity of the far-UVC field is described through the mono-energetic, fixed source Boltzmann transport equation,

2 $$\begin{aligned} \varvec{\Omega }\cdot {\varvec{\nabla }} E({\varvec{r}},\varvec{\Omega })+\Sigma _{t}({\varvec{r}}) E({\varvec{r}},\varvec{\Omega }) - \int _{\varvec{\Omega }'} \Sigma _{s}({\varvec{r}},\varvec{\Omega }' \rightarrow \varvec{\Omega }) E({\varvec{r}},\varvec{\Omega }') d \varvec{\Omega }' = S({\varvec{r}}, \varvec{\Omega }). \end{aligned}$$

The radiation intensity distribution $E({\varvec{r}},\varvec{\Omega })$ exists within a 5 dimensional phase-space consisting of 3 space dimensions, ${\varvec{r}}$, and 2 in angle $\varvec{\Omega }$, with units ${\text {mJ}}.{\text {cm}}^{-2}. {\text {s}}^{-1}$. The equation describes the transport of far-UVC photon energy and includes the photon interaction with their surrounding media through absorption and scattering which are characterised by the cross-sections $\Sigma _t({\varvec{r}})$ and $\Sigma _s({\varvec{r}})$, respectively. The source of far-UVC emanating from a lamp is described through the term $S({\varvec{r}}, \varvec{\Omega })$.

The solution to Eq. (2) was obtained via a model using discontinuous finite elements and discrete ordinates for resolving the spatial and angular dimensions respectively. The solutions presented here used a uniform mesh of $150 \times 150$ quadrilateral elements with linear basis functions. A high order $S_{80}$ angular discretisation was employed to resolve the direction of photon travel. In 2D this used 3280 directions which provided sufficient resolution to cover the whole room with far-UVC with reduced oscillations from ray-effects. This space-angle discretisation resulted in a total of around 295 million degrees of freedom for the whole radiation solution.

The scalar quantity of the spatially dependent far-UVC intensity, $E_p({\varvec{r}})$, that irradiates airborne virus was obtained by integrating over the angular dimension of the intensity variable,

3 $$\begin{aligned} E_p({\varvec{r}}) = \int _{\varvec{\Omega }} E({\varvec{r}},\varvec{\Omega }) d \varvec{\Omega }. \end{aligned}$$

The material cross-sections were derived from a number of sources and was based on dry air, these are summarised in Table 1.

### Fluid flow model for room ventilation

Computational fluid dynamics is a numerical approach for simulating the movement of air based on the conservation laws of mass, momentum, and energy. Ignoring the temperature influences, the airflow motion is governed by the following form of the unsteady, incompressible Navier-Stokes equations:

4 $$\begin{aligned}&{\varvec{\nabla }} \cdot {\varvec{u}} = 0 ,\\&{\varvec{u}}_t+{\varvec{u}}\cdot {\varvec{\nabla }}{\varvec{u}} + {\varvec{\nabla }} p - \nu {\nabla }^2 {\varvec{u}} = 0 . \end{aligned}$$

The velocity of air is denoted by the 3 component vector ${\varvec{u}}=(u,v,w)$ which holds the respective air velocities in the x, y and z dimensions, and *p* denotes the pressure. The kinematic viscosity of air is denoted by $\nu$ and has the value $1.5 \times 10^{-5}\,{\text {m}}^2.{\text {s}}$. With room side lengths of $3{\text {m}}$ and with inlet velocity $0.1 \,{\text {m}}\,{\text {s}}^{-1}$, for 8 ACH ventilation, the Reynolds number ($Re=\frac{U L}{\nu }$ ) for this problem was approximately 30,000.

In the simulations presented a finite element discretisation of the governing equations (4) was used^31^. A regular mesh of $300 \times 300$ quadrilateral elements was employed upon which both the velocities and pressures were resolved using continuous linear basis functions. The transient process was resolved using the explicit Adams–Bashforth stepping scheme. A Large Eddy Simulation was embedded in the fluid solver for resolving the flows’ turbulent features. The full details of the finite element discretisation of the equations (4–5) and the LES model are discussed in^31^.

### UVC inactivation model

The distribution and transportation of the airborne virus was included in the room ventilation model. The spatially dependent scalar concentration of the virus was described through the equation,

5 $$\begin{aligned} (\phi _t+ {\varvec{u}}\cdot {\varvec{\nabla }}\phi ) = \nabla ^2 D \phi + S_{\phi } - ZE_p\phi - \alpha \phi . \end{aligned}$$

The variable $\phi$ denotes the concentration of virus per unit volume ($\hbox {pfu.cm}^{-3}$) which is transported through convection with the air flow ${\varvec{u}}$ and via diffusion with coefficient D. The SARS-CoV-2 source is defined by $S_{\phi }$, and its removal is defined through the last term of Eq. (5). This removal accounts for the inactivation due to the far-UVC intensity field $E_p$, with Z being the far-UVC susceptibility constant. The natural death rate, or half life of SARS-CoV-2 has been considered in the model. The decay rate $\alpha$ is estimated by the reported virus half life of approximately 1.2 h in aerosols^26^.

In the results presented the same spatial and temporal discretisation as the fluid model were used. The far-UVC intensity field in Eq. (3), which was resolved on a different mesh, was conservatively mapped onto the fluids mesh to enable the calculation of viral removal.

The use of Eq. (5) implies the model is concerned with the virus contained within those droplets sufficiently small to remain airborne for periods lasting 10’s of minutes. Thus the larger droplets heavily influenced by gravity and which fall to ground are not considered here. Settling velocities, with typical values of 0.06-$0.35 \,{\text {cm}}. {\text {s}}^{-1}$^32^, and evaporation of droplets have also been omitted from consideration. The droplet’s convection with the air flow is the dominant transport process, and so gravitational effects are small, and any size reduction due to evaporation increases this effect. The resting of droplets on surfaces are currently not included in this model as the analysis centres on the droplets that remain airborne. However, the percentage of those droplets that do come to rest will still be subjected to far-UVC irradiation, but will not be removed through ventilation. Therefore the estimates of removal via the far-UVC are conservative, and the true removal rates are potentially greater.

### Physical properties and model parameters

Table 1 lists all the physical properties and parameters used in the numerical models. The bottom left and top right corners of the bed, head and far-UVC source are located at positions (in m) (1.0, 0.4) and (2.0, 0.7), (1.4,0.6) and (1.6, 0.9), (2.8, 2.8) and (3.0, 3.0), respectively.

**Table 1:** Physical properties and parameters in the numerical experiments.

Symbol	Description	Units	Example case
$\lambda$	Far-UVC wavelength	nm	222
*S*	Far-UVC source	${\text {mJ}}.{\text {cm}}^{-2}. {\text {s}}^{-1}$	0.0022
$\Sigma _t$	Absorption cross section of air	${\text {cm}}^{-1}$	$2.83 \times 10^{-5}$
$\Sigma _s$	Scattering cross section of air	${\text {cm}}^{-1}$	$4.6 \times 10^{-6}$
$\nu$	Air kinematic viscosity	${\text {m}}^2\,\hbox {s}^{-1}$	$1.5 \times 10^{-5}$
D	Diffusion coefficient	${\text {m}}^2\,\hbox {s}^{-1}$	$1.0 \times 10^{-3}$
v	Ventilation inlet flow velocity	$\hbox {m} \,\hbox {s}^{-1}$	$0.01 - 0.1$
ACH	Air change per hour	None	$0.8 - 8.0$
Z	Virus UVC susceptibility constant	$\hbox {cm}^2\,\hbox {mJ}^{-1}$	4.1
$\alpha$	SARS-CoV-2 decay rate in aerosols	None	$1.6 \times 10^{-4}$
L	Room width and height	m	3.0

## Data Availability

Source data files are provided with this paper for Figs. 1–4. at: https://github.com/agbuchan/UVCdata.
